# Impact of Subtalar Distraction Arthrodesis on Ankle Joint: Radiological Insights from Modified Grice–Green Procedure

**DOI:** 10.3390/life14060692

**Published:** 2024-05-28

**Authors:** Elena Artioli, Antonio Mazzotti, Edoardo Cassanelli, Laura Langone, Michele Astolfi, Pejman Abdi, Simone Ottavio Zielli, Alberto Arceri, Cesare Faldini

**Affiliations:** 1 1st Orthopaedics and Traumatologic Clinic, IRCCS Istituto Ortopedico Rizzoli, 40136 Bologna, Italyedoardo.cassanelli@ior.it (E.C.); laura.langone@ior.it (L.L.); pejman.abdi@ior.it (P.A.); simoneottavio.zielli@ior.it (S.O.Z.); alberto.arceri@ior.it (A.A.);; 2 Department of Biomedical and Neuromotor Sciences (DIBINEM), Alma Mater Studiorum University of Bologna, 40123 Bologna, Italy; 3 Department of Neuroscience and Rehabilitation, University of Ferrara, 44121 Ferrara, Italy; stlmhl@unife.it

**Keywords:** subtalar arthrodesis, progressive collapsing foot deformity, ankle, talar tilt angle, arthritis, Grice–Green procedure

## Abstract

Subtalar distraction arthrodesis (SDA) is a surgical procedure designed to treat hindfoot deformities associated with isolated subtalar joint arthritis. In 1996, Fitzgibbons was the first to observe that, in some cases, hindfoot fusion appeared to be associated with the development of tibiotalar valgus tilt. Since then, few studies have addressed this issue. Given that hindfoot fusion can be performed using various techniques, this study investigated the potential tibiotalar joint frontal or sagittal modifications resulting from the modified Grice–Green technique. All the consecutive patients who underwent the modified Grice–Green procedure were included. The patient records were reviewed to extract demographic data. Weight-bearing foot and ankle radiographs were assessed to measure the talar tilt angle and the tibiotalar ratio on the same picture archiving and communication system by three independent observers. A total of 69 patients met the criteria for inclusion. The mean talar tilt showed no substantial changes, since the increase from 1.46 ± 1.62 preoperatively to 1.93 ± 2.19 at a minimum of 8 months postoperatively was not statistically significant (*p* = 0.47). The average preoperative tibiotalar ratio significantly increased from 33.4 ± 4.4% to 35 ± 4% postoperatively (*p* = 0.007), although remaining within the normal range, indicating a possible realignment of the posterior facet of the subtalar joint. In conclusion, this study highlights the effectiveness of the modified Grice–Green procedure in achieving a favorable realignment without impacting the ankle joint, particularly regarding tibiotalar valgus tilt.

## 1. Introduction

Subtalar distraction arthrodesis (SDA) is a surgical intervention employed to treat hindfoot deformities with isolated subtalar joint arthritis. The objectives of this procedure encompass several critical goals: to reclaim hindfoot height by restoring the correct talocalcaneal relationships, rectify heel width by eliminating any fibular impingement, and effectively manage subtalar joint arthritis by fusing the joint. This approach, aimed at correcting the deformity, reduces patient pain and improves functionality [1].

The indications for SDA reflect a spectrum of conditions characterized by subtalar joint arthritis or rigid deformity. Among the diverse etiologies recognized are progressive collapsing foot deformity, calcaneal fractures sequelae, Charcot neuroarthropathy, degenerative and rheumatoid arthritis, subtalar instability, congenital conditions such as talocalcaneal coalition, and residual or overcorrected clubfoot deformities. All these factors can collectively contribute to subtalar joint arthritis or rigid deformity of the hindfoot in the valgus, or both. Consequently, the multifactorial nature of these conditions underscores the importance of SDA as a therapeutic option in managing various subtalar joint pathologies, as it is capable of addressing both deformity and arthritis [1,2,3,4].

Numerous techniques have been described for SDA, displaying variations across several critical dimensions. These encompass the positioning of the patient during the procedure, which may entail prone, lateral, or supine positioning, depending on the surgical approach preferred by the surgeon. The lateral and supine positions are usually used for the lateral approach, whereas prone positioning may be more comfortable for the posterior approach. Additionally, variability is evident in the surgical approach adopted, ranging from lateral to posterior. Moreover, the choice of bone graft material represents another pivotal variable in the procedure. The options include both allograft and autograft sourced from the proximal tibia or iliac crest. This may depend on the surgeon’s preference, the availability of bone banks, and the possibility of performing autologous grafting. Autografting has the advantage of high fusion success rates but carries the disadvantage of potential donor site complications, so some surgeons prefer allografting despite the higher risk of nonunion [5,6]. Furthermore, the method of fixation employed constitutes a crucial aspect of SDA. The fixation methods may entail the use of single or multiple screws, as well as Kirschner wires. The use of screws allows for compression at the arthrodesis site, while K-wires have the advantage of not leaving permanent fixation devices [1,2,7,8].

Among these techniques, the Grice–Green SDA stands as a widely accepted procedure, recognized for its efficacy and versatility in addressing subtalar joint pathologies. Initially described in 1952 by Grice, several modifications have since been introduced to enhance the original technique [2,9,10,11]. Good clinical and functional outcomes have been achieved through these modifications. The outcomes of the modified Grice–Green SDA have consistently shown promising results, with a reported union rate averaging 96% overall. Furthermore, the modified American Orthopaedic Foot and Ankle Society (AOFAS) score typically reaches 73 out of 94 points postoperatively, reflecting an average increase of 44.2 points compared to the preoperative scores, underscoring the procedure’s effectiveness in enhancing patient function and quality of life. Minor complications were noted in around 5% of cases, predominantly comprising wound complications. Additionally, less frequently observed complications included malunion or secondary dislocation, sural neuritis, and complex regional pain syndrome (CRPS) [8]. This study focuses on potential modifications to the tibiotalar joint’s alignment resulting from the modified Grice–Green technique, an area that has not previously been assessed.

The clinical and radiological outcomes of SDA have been extensively analyzed in the literature. Despite this, the influence of SDA on the angular parameters of the tibiotalar joint has rarely been evaluated [8]. This is despite Fitzgibbons’ observation in 1996 that hindfoot fusion appeared in some cases to be associated with the development of tibiotalar valgus tilt [12]. Since then, only two studies have addressed this issue, providing limited insights into its complexities and implications [13,14].

This topic is therefore still in need of further investigation, including the evaluation of potential differences between various SDA techniques. Therefore, the objective of this study is to investigate the potential tibiotalar joint frontal or sagittal modifications resulting from the modified Grice–Green technique, with a specific focus on assessing any potential changes in anatomical alignment.

## 2. Materials and Methods

This study was conducted according to the guidelines of the Declaration of Helsinki, and it was approved by the Ethics Committee of Rizzoli Orthopedic Institute (protocol code 369/2022/Oss/IOR; date of approval: 19 May 2022). Each patient, recognizing the significance of their participation, willingly provided informed and signed consent for inclusion in the study.

The study included all the patients consecutively enrolled for the modified Grice–Green SDA procedure for progressive collapsing foot deformity from 2021 to 2023.

The inclusion criteria encompassed adult patients affected by progressive collapsing foot deformity who underwent the modified Grice–Green SDA procedure, were capable of adhering to the postoperative protocol, had undergone only Achilles tendon lengthening as an additional procedure, and had available full weight-bearing foot and ankle radiographs preoperatively and postoperatively, with a minimum follow-up of 8 months. Patients under the age of 18, those with a history of ankle fracture or previous ankle surgeries, additional procedures other than isolated Achilles tendon lengthening, revisions of subtalar arthrodesis, and patients with missing radiological data were excluded.

**Figure 1:**
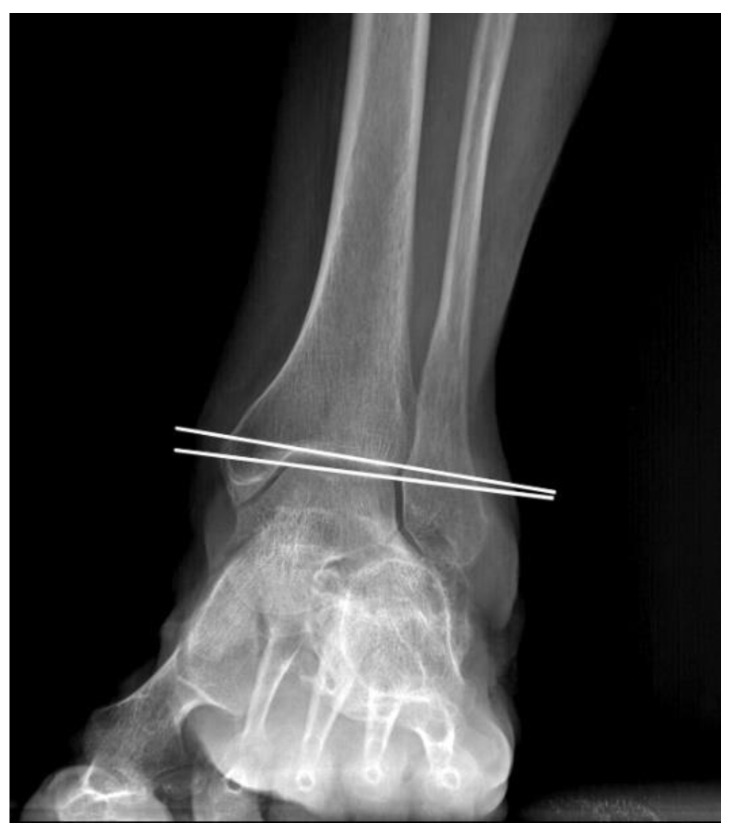
Talar tilt measurement, defined as the angle formed between the articular surface of the talus and the tibial plafond.

**Figure 2:**
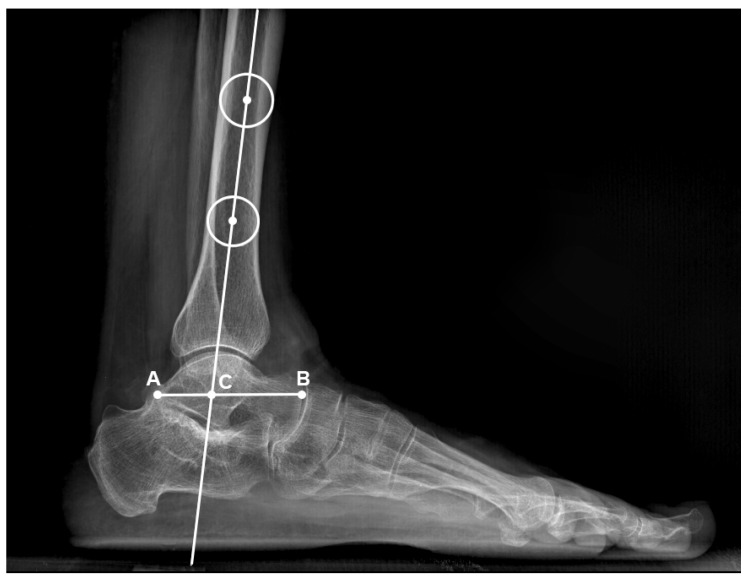
Tibiotalar ratio measurement, defined as the ratio between the length of the talus segment posterior to the tibial axis and the overall longitudinal length of the talus in lateral view. The tibiotalar ratio is defined as the ratio between the AC and AB.

The patient records were reviewed to extract demographic data, encompassing age, gender, surgical indication, follow-up duration, and any additional procedures undertaken during the surgical treatment. Full weight-bearing foot and ankle radiographs of all the included patients were assessed to measure the following:

- The talar tilt (Figure 1): Defined as the angle formed between the articular surface of the talus and the tibial plafond on the mortise or anteroposterior view. The normal value described in the literature ranges below 2 degrees [15].
- The tibiotalar ratio (Figure 2): Defined as the ratio between the length of the talus segment posterior to the tibial axis and the overall longitudinal length of the talus in lateral view. The normal value is reported in the literature to be 33.4% ± 3.3% [16].

The measurements were conducted independently by three residents trained in foot and ankle pathology, each blinded to the measurements of the others on the same picture archiving and communication system (Carestream Health, Inc, Rochester, NY, USA). To ensure consistency in the measurements, the presence of an os trigonum or of a Stieda process was excluded from the calculation of the tibiotalar ratio.

Considering the studies published so far evaluating the sensitivity of radiological parameters to changes in ankle positioning, postoperative changes in ankle alignment were defined as a variation of at least 2 degrees in the talar tilt and a variation of at least 2% in the tibiotalar ratio compared to the preoperative measurements [16].

### 2.1. Surgical Procedure

All the patients were operated on by two experienced foot and ankle orthopedic surgeons. The patient was positioned in a supine position. A tourniquet was applied at the root of the thigh to minimize intraoperative bleeding. The dorsiflexion of the ankle joint was evaluated: in the case of Achilles tendon tension, Achilles tendon lengthening was performed using two alternating half-sections, according to White [17].

A lateral incision of approximately 3 cm at the sinus tarsi level was performed. The fat pad was removed to allow visualization of the subtalar joint. Through specific osteotomies, the posterior subtalar joint was debrided until exposing subchondral spongy bone at both the calcaneal and talar surfaces, where microperforations were made. The talocalcaneal relationships were manually reduced and stabilized using a retrograde 2 mm K-wire from the calcaneus to the talus under fluoroscopic control. Using a chisel of 2 cm, a slot was prepared between the talus and the calcaneus, just anteriorly to the lateral process of the talus, aligned with the tibial axis. A medial approach to the proximal tibia was performed to harvest, with an oscillating saw, a 2 cm × 1 cm corticospongious bone graft. The cortical portion of the bone graft was positioned in the previously prepared slot, while the spongy component was interposed between the debrided surfaces of the posterior subtalar joint to facilitate fusion. It was therefore ensured that the cortical graft did not create any lateral impingement with the fibula. The tourniquet was released to achieve accurate hemostasis. Abundant irrigation was carried out during the surgery. Closure was performed in layers using absorbable suture [2].

The postoperative course involved the application of a plaster cast on the operated limb, maintained for 40 days without weight-bearing. After 40 days, the plaster boot was removed, follow-up X-rays were taken, and the calcaneal K-wire was removed. Subsequently, another plaster boot was applied, allowing progressive weight-bearing with the aid of two crutches for an additional 35 days. At the end of this period, further follow-up X-rays were taken, and the plaster boot was replaced with a comfortable shoe. The patients remained under thromboembolic prophylaxis until achieving complete weight-bearing in the shoe.

### 2.2. Statistical Techniques

The data involved in this work were presented by the mean values together with their standard deviations. Since three independent raters, residents trained in foot and ankle pathology, were recruited to carefully estimate both the talar tilt angle and tibiotalar ratio of the same patient pool, the inter-rater agreement among the data was evaluated by using the intraclass correlation coefficients (ICCs) derived from a two-way random model [18].

The standard error of measurement (SEM) was computed following the relation: $SEM=\sigma \cdot \sqrt{\left(1-ICC\right)}$, where *σ* is the standard deviation. The use of the SEM is more appropriate in this context, considering its dependence on the ICC, indicating how reliable the recorded data were. Normality was investigated through the widely used Shapiro–Wilk test; it is a very powerful test suitable for evaluating normality in even very small samples by comparing a non-parametric and a parametric estimator of the sample variance [19]. Levene’s test was performed to determine if two (or more) samples had or not an equal variance. This step was crucial to choose the proper statistical test to apply to the dataset since many significance tests must be performed on only samples exhibiting the same variance, such as the *t*-test. Paired *t*-tests (parametric) were employed to compare the preoperative and follow-up values for those data normally distributed and showing the same variance, while for the ones non-normally distributed the Wilcoxon test (non-parametric test) was adopted. Finally, the correlation between the data was determined by using both the Pearson and Spearman coefficients. The latter were used in parallel as a double-check about the correlation: though the Pearson coefficient requires the data normality (sometimes not satisfied) and the absence of outliers in the sample, it can also be used with good reliability for those data non-normally distributed; the Spearman coefficient is used as a robust tool to assess the correlation between two normally or non-normally distributed and monotonic data groups, also comprising possible outliers [20].

Statistical significance was predetermined at a *p*-value of less than 0.05. All the statistical analyses were performed by using the SciPy Python Library (https://scipy.org/ accessed on 23 April 2024).

## 3. Results

### 3.1. Population

During the expansive period spanning from 2021 to 2023, a total of 98 patients affected by progressive collapsing foot deformity underwent the modified Grice–Green SDA surgical technique. However, 11 patients were excluded from the analysis due to incomplete radiological data, which precluded complete calculations, while an additional 12 patients were excluded due to a follow-up duration of less than 8 months. Moreover, six patients were excluded as they underwent additional procedures whose effects on ankle alignment have not been evaluated, including removal of a subtalar screw, correction of hallux valgus, arthrodesis of the proximal interphalangeal joint of the second toe, accessory scaphoid excision, Morton’s neuroma excision, and excision of talocalcaneal synostosis.

**Figure 3:**
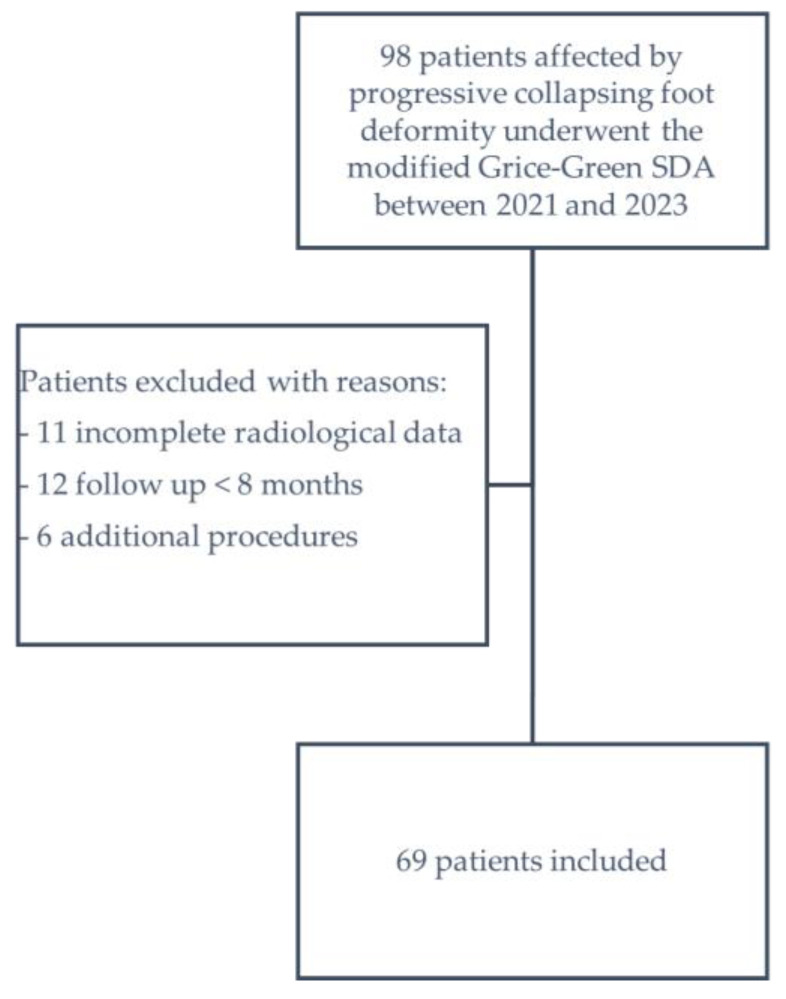
Graphical representation of the patient selection process.

Following a rigorous patient selection process characterized by scrupulous adherence to predefined criteria, a notable cohort comprising 69 patients emerged as eligible candidates for inclusion in the study (Figure 3). This group consisted of 42 females and 27 males. The mean age at the time of surgery was calculated to be 52 ± 14 years. Additionally, the included patients exhibited a mean body mass index (BMI) of 27 ± 4. A total of 35 patients (47%) underwent surgery on the right foot, while the remaining 39 patients (53%) on the left foot. A total of 30 patients (43%) underwent Achilles tendon lengthening as an additional procedure with alternate emisections.

Only one operated patient developed a graft mobilization complication, necessitating reintervention to remove a lateral peroneal impingement. Additionally, two patients underwent reoperation, one for total ankle replacement and the other for the removal of an anterior tibiotalar impingement (Table 1). However, the radiological values were measured on the latest radiographs available before the reoperation to ensure that the results of the study were not impacted.

### 3.2. Inter-Rater Agreement

The ICCs (df = 68,136) regarding the preoperative (TT0) and postoperative (TT8) talar tilt measurements, performed by the three independent and equally trained raters, highlighted the high agreement between raters, proving to be 0.95 (CI: 0.92–0.97) and 0.97 (CI: 0.95–0.98), respectively. Similar ICC (df = 68,136) values were obtained for the preoperative (TR0) and postoperative (TR8) tibiotalar ratio, resulting in 0.91 (CI: 0.86–0.94) and 0.85 (CI: 0.78–0.90), respectively. Although these ICC values were a little bit lower than the TT ones, they still show a very good agreement among raters. To further investigate the agreement and reliability between the raters’ measurements, the ICC was also computed on the TT and TR variations between the pre- and postoperative values (i.e., TT8–TT0 and TR8–TR0 data): the computed ICCs were 0.95 (CI: 0.93–0.97) and 0.86 (CI: 0.8–0.91), respectively. Since all the ICCs computed were significantly above 0.8, the measurements were reliable and hence the raters were in a very good agreement with each other.

### 3.3. Talar Tilt

Among the cohort of 69 patients, 10 individuals (14.5%) exhibited a change in the talar tilt angle. In more detail, the talar tilt angle increased in 9 patients and decreased in 1.

Since the Shapiro–Wilk normality test performed on the two datasets, TT0 and TT8, showed that none of them was normally distributed (both *p*-values << 0.05), the Wilcoxon test was applied to the datasets. TT0 and TT8 were found to be equally distributed (Wilcoxon’s test *p*-value of 0.47), both with almost the same variance, as computed by using Levene’s test with a *p*-value of 0.21. Lastly, it is worth considering that the mean preoperative talar tilt was 1.46 ± 1.62, increasing to 1.93 ± 2.19 after at least 8 months from the surgical treatment (Table 2). Moreover, eight out of the ten patients who experienced a significant change in the talar tilt angle were demonstrated to have a final talar tilt angle in the valgus, and among these eight subjects, four already had a valgus talar tilt preoperatively (as inferred from the TT0 dataset). Three out of these four cases worsened the talar tilt angle and one of them improved it, albeit remaining beyond the physiological 2 degrees.

### 3.4. Tibiotalar Ratio

For what concerns the tibiotalar ratio parameter, the TR0 average proved to be 33.4 ± 4.4%, and it increased to 35.0 ± 4.0% after the surgical treatment. As performed for the talar tilt, the normality was tested at first by using the Shapiro–Wilk test: while TR8 proved to be normally distributed (*p*-value of 0.04), the TR0 dataset was not normally distributed (*p*-value of 0.55). Based on this observation, given that at least one of the datasets did not exhibit a normal distribution, the Wilcoxon test was selected for comparing the two datasets. The results of the analysis revealed a significant difference between the distributions of the two datasets, as indicated by a Wilcoxon’s test *p*-value of 0.007 (Table 2).

### 3.5. Correlation between Talar Tilt and Tibiotalar Ratio

Given that the talar tilt and tibiotalar ratio variations are two parameters that could potentially exhibit correlation with each other, a comprehensive correlation analysis was conducted. This analysis utilized both Pearson’s and Spearman’s correlation coefficients to thoroughly explore the relationship between these variables. The two datasets were found to be completely uncorrelated since the Pearson’s and Spearman’s correlation coefficients were −0.02, and −0.03, respectively.

**Figure 4:**
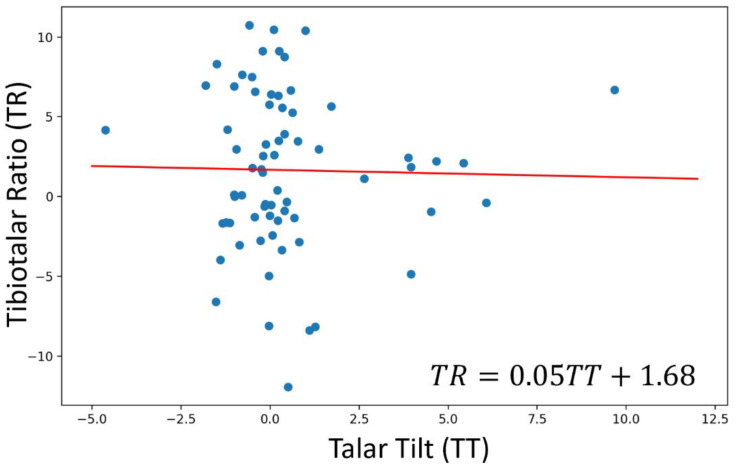
Dispersion plot with the regression line of talar tilt vs. tibiotalar ratio variations. Blue dots were obtained by plotting the patient’s tibiotalar ratio variations (*y* axis) as a function of their talar tilt ones (*x* axis). The simple linear regression was performed on the data and the resulting regression line was reported in red. Since this line is very close to horizontality (angular coefficient = 0.05, interceptor = 1.68, and r^2^ = 3.9 × 10^−4^), it is reasonable to infer that there is no correlation between these two variables.

To further investigate the possible correlation between the talar tilt and tibiotalar ratio variations, besides the Pearson’s and Spearman’s correlation coefficients, a simple linear regression model was performed on the dataset. As shown in the dispersion plot (Figure 4), the regression red line was very close to horizontality (r^2^ = 3.9 × 10^−4^; *y* = 5 × 10^−2^*x* + 1.68), indicating the almost complete uncorrelation between a patient’s tibiotalar ratio and talar tilt variations within the eight months after surgery.

## 4. Discussion

SDA stands as a widely employed surgical intervention, renowned for its remarkable efficacy in reinstating optimal talo-calcaneal alignment. Recent scrutiny by select researchers has unveiled the possibility of subtalar intervention exerting consequential effects on the articulatory dynamics at the tibiotalar interface [13,14]. Given the array of surgical methodologies available for executing subtalar fusion, the primary objective of this study was to evaluate whether the modified Grice–Green SDA engendered alterations in the tibiotalar relationships. Such information could hold profound significance for the scientific community, offering the potential for a nuanced reassessment of the procedural utility and the requisite consideration of adjunctive measures in specific patient cohorts.

The present investigation adopted a retrospective monocentric design. To mitigate potential sources of bias in the absence of pertinent literature precedents, the inclusion criteria were very stringent.

All the patients who underwent additional procedures were systematically excluded, with the only accepted adjunctive procedure being Achilles tendon lengthening.

The necessity of Achilles tendon lengthening frequently emerges in the correction of flatfoot deformities via the modified Grice–Green SDA owing to the commonplace observation of Achilles retraction following hindfoot realignment [21]. Its pervasiveness was also confirmed by its incorporation in 43% of cases within this study.

However, the non-exclusion of Achilles tendon lengthening will not influence the results of this study, as supported by a cadaveric research demonstrating that Achilles lengthening does not result in a change in the mean pressure, peak pressure, or center of force contact area in the ankle [22]. Therefore, the decision was made to include patients who underwent only Achilles tendon lengthening as an additional procedure in order to prevent the loss of a substantial portion of operated patients, and also considering the demonstrated lack of influence of this procedure on the angular values of the tibiotalar joint.

Additionally, a minimum follow-up period of 8 months was selectively adopted for patient inclusion. This choice was informed by previous investigations, which revealed the emergence of postoperative valgus tibiotalar tilt at mean follow-up intervals of 3.6 months per Miniaci-Coxhead et al. and 7.7 months per Kim et al. [13,14]. In a bid to achieve meticulousness, a conservative threshold of 8 months was established to ensure comprehensive assessment and to navigate potential fluctuations in postoperative outcomes.

The findings derived from this study regarding the impact of the modified Grice–Green SDA procedure on the tibiotalar relationship have proven remarkably intriguing.

In contrast to findings reported by previous authors [13,14], the modified Grice–Green SDA did not appear to correlate with the occurrence of valgus talar tilt.

In the research conducted by Kim et al. involving a cohort comprising 59 patients, the findings revealed that 28.8% of the subjects exhibited the development of postoperative valgus talar tilt. Moreover, the study elucidated a statistically significant association between a heightened degree of hindfoot valgus and the incidence of valgus talar tilt following the surgical intervention [13]. A similar percentage was also observed by Miniaci-Coxhead et al. In their study involving 187 patients, 27.3% developed a postoperative valgus tibiotalar tilt. Interestingly, this study revealed an association between an increased Meary angle and the onset of ankle deformity [14]. Some differences need to be highlighted compared to other studies. In this work, only SDAs were considered, whereas Miniaci-Coxhead et al. also included triple arthrodeses, which constituted most of the interventions in their study. Additionally, it is noteworthy that more than half of their patients with valgus talar tilt (27 out of 51) already had a preoperative tilt. Differently, in this study, only patients who had a change of at least 2° from the pre- to postoperative state were examined; otherwise, SDA was not considered to be the cause of a pre-existing valgus tilt [14]. Kim et al., on the other hand, excluded all patients with preoperative valgus tilt, and all the included patients underwent a subtalar fusion. The significant difference compared to the present study is that many additional procedures associated with subtalar fusion were considered, whose influence on talar tilt has never been analyzed [13].

In the present study, a slight increase in talar tilt was observed in 13% of patients, with no statistically significant difference noted in the pre- and postoperative values. Conversely, a decrease in the talar tilt angle was observed in 1.4% of cases. Within the cohort of 10 patients experiencing a modification to the talar tilt, eight showed a postoperative valgus, but four of these already had it before the surgery. Thus, a total of four (5.8%) patients developed a valgus talar tilt following the modified Grice–Green SDA. It is pertinent to highlight that within the preoperative cohort, a total of 20 patients manifested a pre-existing valgus talar tilt (exceeding 2 degrees). Of these individuals, only 3 exhibited a postoperative deterioration in their condition, while 17 demonstrated a discernible improvement. Notably, one patient within this group retained the valgus tilt despite the improvement.

Therefore, based on the results of this study, it cannot be assumed that the modified Grice–Green SDA is associated with the onset or exacerbation of valgus talar tilt, even in cases where it was already present preoperatively.

Furthermore, it is noteworthy that both the preoperative and postoperative mean values consistently fell within the normal range, indicating stability and alignment within the physiological parameters throughout the course of the study.

This was nonetheless predictable, given that only patients who underwent an SDA without additional procedures were included. A preoperative valgus talar tilt is usually associated with deltoid ligament insufficiency, which is present in stage IV of adult flatfoot, according to Myerson [23]. This condition typically requires additional procedures [24]. Therefore, the exclusion criteria also indirectly excluded patients with severe valgus of the tibiotalar joint.

Similarly, the pre- and postoperative tibiotalar ratio values were observed to consistently remain within the normal range throughout the duration of the study. Notably, the increase in the tibiotalar ratio observed at 8 months postoperatively was found to be statistically significant. This outcome can be plausibly attributed to the corrective realignment of the talus and calcaneus facilitated by the modified Grice–Green SDA procedure. This surgical intervention is particularly efficacious in addressing progressive collapsing foot deformity, which is characterized by a medial collapse of the talus. Such collapse may result in diminished coverage of the posterior facet of the subtalar joint compared to healthy individuals [25]. Through the modified Grice–Green SDA procedure, the restoration of proper talo-calcaneal relationships is achieved. This corrective measure enables a posterior repositioning of the talus relative to the calcaneus, thereby rectifying the malalignment characteristic of the progressive collapsing foot deformity. As a consequence of this realignment, a slight increase in the tibiotalar ratio is observed postoperatively. Importantly, despite this observed increase in the tibiotalar ratio, it is noteworthy that the values remained well within the normal range. This result is clinically reassuring because the presence of talolisthesis can be associated with end-stage ankle arthritis, a condition that should not be present in the patients included in this study [26]. Analyzing the radiological values of the patient who later required ankle replacement, a slight valgus tibiotalar tilt and a slight anterior talus displacement were noted, even preoperatively. While a single case cannot be generalized, it underscores the importance of evaluating these radiological values. Long-term studies are necessary to determine if a moderate alteration in the frontal or sagittal alignment in patients with progressive collapsing foot deformity undergoing SDA might suggest the need for future ankle surgeries.

Finally, while the assessment of the safety profile of this procedure was not the primary focus of this study, it is remarkable to observe that the modified Grice–Green procedure exhibited favorable results and was associated with a low incidence of complications.

### Limitations

The foremost limitation of this study pertains to the relatively minute angular values characterizing the tibiotalar tilt. It is imperative to acknowledge that a mere 1–2-degree measurement error could exert a considerable influence on the overarching study outcomes. Given that a change of just 2° in the talar tilt was considered significant, a measurement error of 2° would compromise the results because it would be impossible to determine whether a 2° change is due to an actual alteration or a measurement error. To mitigate this problem, it is necessary to achieve the requisite measurement precision, which demands an exceedingly meticulous measurement system—a feat not easily attainable with standard AP radiographic imaging. Despite the commendable inter-rater correlation observed among the three assessors, substantial challenges were encountered during the practical execution of the measurements, particularly in delineating the precise points for the tibiotalar tilt assessment. Notably, the tibial plafond often lacks distinct demarcation on AP radiographs, frequently presenting overlapping structures that obscure unequivocal identification. Consequently, prior to initiating measurements, a consensus was reached with the senior author regarding the selection of pertinent landmarks for defining the tibial plafond line. Conversely, the identification of the talus articular surface proved comparably more straightforward. Furthermore, in the measurement of the TT ratio, any presence of os trigonum or Stieda processes, which could potentially distort the veracity of measurements, was not taken into consideration.

Another limitation stems from the relatively modest sample size enrolled in the study, which can be attributed to the stringent nature of the inclusion and exclusion criteria. However, the decision to uphold stringent criteria for patient inclusion aimed to mitigate potential biases and ensure the validity of the findings. Given the paucity of the literature on this topic, uncertainties persist regarding the factors that may influence the measurements obtained. Therefore, stringent inclusion criteria were deemed imperative to enhance the credibility and robustness of the study outcomes.

The relatively brief average follow-up period is unlikely to be a limitation. As previously discussed, the literature suggests that the development of valgus tibiotalar tilt occurs within an 8-month timeframe, and the follow-up duration within the present study exceeds this critical threshold.

Finally, a potential source of bias was the significant variability in radiological values among the patients. This was evidenced by the fact that the standard deviations of both the talar tilt and the tibiotalar ratio were often higher than their means. This demonstrates that the dataset exhibited considerable variability. Thus, studies on more homogeneous populations might yield more specific results, though these would be less generalizable.

## 5. Conclusions

The results of this study have demonstrated that the modified Grice–Green SDA procedure is not associated with the onset of tibiotalar joint frontal or sagittal modifications evaluated with the talar tilt angle and tibiotalar ratio. This technique can increase the tibiotalar ratio values, which remain within the normal range. This modification is likely a consequence of restoring the physiological coverage of the posterior facet of the subtalar joint by reinstating normal talo-calcaneal relationships. Restoring the normal joint relationships could improve the joint biomechanics, leading to clinical and functional improvement. Conversely, no statistically significant influence was observed on the talar tilt angle, which remained within physiological ranges both pre- and postoperatively. This outcome was expected, as selecting patients who underwent only SDA excluded stage IV adult flatfoot requiring additional procedures, which may also have ankle alterations.

Significant preoperative and postoperative variability in the radiological values could impact these results. Thus, studies investigating the long-term clinical outcomes of the procedure in specific patient demographics, such as only patients with or without preoperative valgus tilt and/or preoperative tibiotalar ratio alteration, might yield more specific results. However, these findings would likely be less generalizable.

In conclusion, this study was characterized by rigorous patient inclusion criteria and a preliminary consensus on how to measure the radiographic parameters in order to reduce the measurement errors and consequently the potential biases. The results, despite the small sample size, emphasized the effectiveness of the modified Grice–Green SDA procedure in achieving favorable realignment without inducing deviations in the ankle joint.

## Figures and Tables

**Table 1:** Demographic data.

	N = 69
Age (mean, SD)	52 ± 14 Years
Sex F:M	42:27
BMI (mean, SD)	27 ± 4
Laterality Right:Left	35:39
Achilles tendon lengthening No:Yes	39:30
Complications	1 graft mobilization
Reoperations	1 total ankle replacement 1 anterior tibiotalar impingement removal

**Table 2:** Preoperative and postoperative radiological parameters.

	Preoperative Mean ± SD	Postoperative Mean ± SD	*p*-Value
Talar tilt	1.46 ± 1.62	1.93 ± 2.19	0.47
Tibiotalar ratio (%)	33.4 ± 4.4	35 ± 4	0.007

## Data Availability

The data presented in this study are available on request from the corresponding author due to privacy, legal and ethical reasons.

## References

[1] Espinosa N., Vacas E. (2018). Subtalar Distraction Arthrodesis. Foot Ankle Clin..

[2] Mosca M., Caravelli S., Vannini F., Pungetti C., Catanese G., Massimi S., Fuiano M., Faldini C., Giannini S. (2019). Mini Bone Block Distraction Subtalar Arthrodesis (SAMBB) in the Management of Acquired Adult Flatfoot with Subtalar Arthritis: A Modification to the Grice–Green Procedure. Joints.

[3] Mann R.A., Beaman D.N., Horton G.A. (1998). Isolated Subtalar Arthrodesis. Foot Ankle Int..

[4] Faldini C., Artioli E., Panciera A., Bonelli S., Arceri A., Mazzotti A. (2023). Surgical Management of Clubfoot Overcorrection: A Case Series. Arch. Orthop. Trauma Surg..

[5] Hollawell S.M. (2012). Allograft Cellular Bone Matrix as an Alternative to Autograft in Hindfoot and Ankle Fusion Procedures. J. Foot Ankle Surg..

[6] McGarvey W.C., Braly W.G. (1996). Bone Graft in Hindfoot Arthrodesis: Allograft vs Autograft. Orthopedics.

[7] Jackson J.B., Jacobson L., Banerjee R., Nickisch F. (2015). Distraction Subtalar Arthrodesis. Foot Ankle Clin..

[8] Schepers T. (2013). The Subtalar Distraction Bone Block Arthrodesis Following the Late Complications of Calcaneal Fractures: A Systematic Review. Foot.

[9] GRICE D.S. (1952). An Extra-Articular Arthrodesis of the Subastragalar Joint for Correction of Paralytic Flat Feet in Children. J. Bone Joint Surg. Am..

[10] Seymour N., Evans D.K. (1968). A Modification of the Grice Subtalar Arthrodesis. J. Bone Joint Surg. Br..

[11] Güven M., Tokyay A., Akman B., Encan M.E., Altintaş F. (2016). Modified Grice-Green Subtalar Arthrodesis Performed Using a Partial Fibular Graft Yields Satisfactory Results in Patients with Cerebral Palsy. J. Pediatr. Orthop. B.

[12] Fitzgibbons T.C. (1996). Valgus Tilting of the Ankle Joint after Subtalar (Hindfoot) Fusion: Complication or Natural Progression of Valgus Hindfoot Deformity?. Orthopedics.

[13] Kim J., Rajan L., Henry J., Mizher R., Johnson A.H., Demetracopoulos C., Ellis S., Deland J. (2023). Incidence and Predictors of Valgus Tibiotalar Tilt after Progressive Collapsing Foot Deformity Reconstruction Using Subtalar Fusion with Concomitant Procedures. Arch. Orthop. Trauma Surg..

[14] Miniaci-Coxhead S.L., Weisenthal B., Ketz J.P., Flemister A.S. (2017). Incidence and Radiographic Predictors of Valgus Tibiotalar Tilt After Hindfoot Fusion. Foot Ankle Int..

[15] Lau B.C., Allahabadi S., Palanca A., Oji D.E. (2022). Understanding Radiographic Measurements Used in Foot and Ankle Surgery. J. Am. Acad. Orthop. Surg..

[16] Tochigi Y., Suh J.S., Amendola A., Pedersen D.R., Saltzman C.L. (2006). Ankle Alignment on Lateral Radiographs. Part 1: Sensitivity of Measures to Perturbations of Ankle Positioning. Foot Ankle Int..

[17] Firth G.B., McMullan M., Chin T., Ma F., Selber P., Eizenberg N., Wolfe R., Graham H.K. (2013). Lengthening of the Gastrocnemius-Soleus Complex: An Anatomical and Biomechanical Study in Human Cadavers. J. Bone Joint Surg. Am..

[18] Koo T.K., Li M.Y. (2016). A Guideline of Selecting and Reporting Intraclass Correlation Coefficients for Reliability Research. J. Chiropr. Med..

[19] Rochon J., Gondan M., Kieser M. (2012). To Test or Not to Test: Preliminary Assessment of Normality When Comparing Two Independent Samples. BMC Med. Res. Methodol..

[20] Rovetta A. (2020). Raiders of the Lost Correlation: A Guide on Using Pearson and Spearman Coefficients to Detect Hidden Correlations in Medical Sciences. Cureus.

[21] Mosier-LaClair S., Pomeroy G., Manoli A. (2001). 2nd Operative Treatment of the Difficult Stage 2 Adult Acquired Flatfoot Deformity. Foot Ankle Clin..

[22] Duggal N., Williamson P., Okajima S., Biggane P., Nasr M., Nazarian A. (2018). Effect of Achilles Tendon Lengthening on Ankle and Subtalar Joint Orientation and Load Distribution Utilizing a Novel Cadaveric Model to Simulate Weight Bearing. Foot Ankle Orthop..

[23] Mann R.A. (1997). Adult Acquired Flatfoot Deformity. Treatment of Dysfunction of the Posterior Tibial Tendon. J. Bone Joint Surg. Am..

[24] Smith J.T., Bluman E.M. (2012). Update on Stage IV Acquired Adult Flatfoot Disorder. When the Deltoid Ligament Becomes Dysfunctional. Foot Ankle Clin..

[25] Knutson K., Peterson A.C., Lisonbee R.J., Hintermann B., Krähenbühl N., Lenz A.L. (2023). Joint Coverage Analysis in Progressive Collapsing Foot Deformity. J. Orthop. Res..

[26] Christensen J.C., Schuberth J.M., Powell E.G. (2016). Talolisthesis in End Stage Ankle Arthrosis. Foot ankle Surg. Off. J. Eur. Soc. Foot Ankle Surg..

